# An *In-Vivo* Study on Anticonvulsant, Anxiolytic, and Sedative-Hypnotic Effects of the Polyphenol-Rich *Thymus Kotschyanus* Extract; Evidence for the Involvement of GABA_A_ Receptors

**DOI:** 10.22037/ijpr.2019.15579.13194

**Published:** 2019

**Authors:** Reza Jahani, Faraz Mojab, Arash Mahboubi, Azadeh Nasiri, Armin Tahamtani, Mehrdad Faizi

**Affiliations:** a *Student Research Committee, Department of Pharmacology and Toxicology, School of Pharmacy, Shahid Beheshti University of Medical Sciences, Tehran, Iran. *; b *Department of Pharmacognosy, School of Pharmacy, Shahid Beheshti University of Medical Sciences, Tehran, Iran. *; c *Food Safty Research Center Department of Pharmaceutics, School of Pharmacy, Shahid Beheshti University of Medical Sciences, Tehran, Iran. *; d *Department of Pharmacology and Toxicology, School of Pharmacy, Shahid Beheshti University of Medical Sciences, Tehran, Iran. *; e *Pharmaceutical Sciences Research Center, Shahid Beheshti University of Medical Sciences, Tehran, Iran.*

**Keywords:** *Thymus kotschyanuse*, GABA-A receptors, Phenolic content, Epilepsy, Insomnia, Memory, Mice

## Abstract

Antidepressant-like activity of *T. kotschyanus* has been recently reported by scientists but insufficient attention has been so far devoted to *T. kotschyanus*, and there is a lack of information on the other neurobehavioral effects and side effects of this species. In the current study, the anticonvulsant, anxiolytic, and sedative-hypnotic, effects of* Thymus kotschyanus* extract on male NMRI mice were evaluated using pentylenetetrazole, maximal electroshock, elevated plus maze, and pentobarbital-induced sleeping tests. Since phenolic compounds and flavonoids have main roles in pharmacological effects of most plant extracts, the phenolic and flavonoid contents of the extract were measured with Folin-Ciocalteu and AlCl_3_ reagents. Acute toxicity, passive avoidance, and open field tests were carried out to assess the toxicity of the extract. To find out the possible mechanism of action, flumazenil as the specific GABA_A_ receptor antagonist was used. Anticonvulsant and hypnotic effects of the extract were observed at 400 and 600 mg/kg. The extract at the dose of 200 mg/kg revealed significant anxiolytic effects, but it did not show any adverse effects on learning and memory at all the tested doses. Results of this study indicate that *Thymus kotschyanus* extract has anticonvulsant‎, anxiolytic, and hypnotic effects, which are likely related to the ability of some phenolic compounds to activate α1-containing GABA_A_ receptors but more experiments still need to be carried out in order to find the exact mechanism, active component, and the toxicity of the* Thymus kotschyanus* extract.

## Introduction

Central nervous system impairment causes both mental and behavior disorders. Epilepsy, unipolar depressive disorders, bipolar affective disorder, schizophrenia, post-traumatic stress disorder, obsessive and compulsive disorder, panic disorder, primary insomnia, Alzheimer’s and other dementias are some of the neuropsychiatric conditions. Mental disorders are not exclusive to a special group of people; they can be found in women and men, different stage of the life, rich and poor people, and among people who live either in urban or rural areas (1). Approximately, 1 in 5 adults in the U.S. (44.7 million, or 18.3%) experiences mental illness in a given year, and almost 1 in 25 adults in the U.S. ( 10.4 million, or 4.2%) experiences a serious mental illness in a given year that substantially interferes with or limits one or more major life activities. Recent studies have revealed this fact that psychiatric symptoms instead of being effects of a common cause probably can cause each other. On the other side, these disorders can lead to other health problems and diseases. For example, insomnia can cause fatigue, feelings of guilt, loss of interest or concentration problems, or anxiety can lead to insomnia. Subsequently, the society will face to a more increased burden of disease compared to a spectacular disease (2). Obviously, economic costs to society and the impacts on the quality of lives are the main burdens of mental disorders (3). Therefore, mental disorders have been considered as a growing public health concern and a major social and economic issue. These problems can affect individuals, families, and societies all over the world (4). 

Herbal medicines have been used for health and medical purposes for several thousands of years because of their lower toxicity and potent ingredients (5). *Thymus kotschyanus* belonging to the *Thymus* genus and *Lamiaceae* family is originated from the Mediterranean area (6). Several biological and pharmacological properties such as antibacterial, antifungal (7), antiviral (8), anti-helminthic (9), anti-oxidative (7, 10), ‎ antispasmodic (11), and sedative (12) effects have been reported for genus *Thymus* in the literature. Antidepressant-like activity of *T. kotschyanus* has been recently reported by researchers (13) but insufficient attention has been so far devoted to *T. kotschyanus*, and there is a lack of information on the other neurobehavioral effects of this species. Therefore, the aim of this study is to evaluate anticonvulsant, anxiolytic, and sedative-hypnotic effects, possible mechanism of action and the toxicity of the *Thymus ‎kotschyanus* extract.

## Experimental

*Plants and extraction*

*Thymus kotschyanus *was obtained from Tabriz province of Iran, and it was authenticated in ‎the Department of Pharmacognosy, School of Pharmacy, Shahid Beheshti University of Medical Sciences (Tehran, Iran). The aerial part of the plant was dried at the room temperature and crushed into a very fine powder by a grinder. The methanolic extract of* T. kotschyanus* was obtained by maceration of the plant powder in 900 mL of methanol 96% on a shaker (Stuart SSL1 shaker, UK) at the room temperature. This procedure was repeated for three consecutive days. Following filtering the solution by a paper filter, the filtrate was concentrated in a rotary evaporator (Heidolph, Germany). The extraction yield (mass of extract/mass of dry matter ×100) was calculated 3.5% w/w.

*Drugs and treatments*

The extract, pentylenetetrazole (Sigma-Aldrich, St. Louis, MO, USA ), pentobarbital (Sigma-Aldrich, St. Louis, MO, USA ) and midazolam (Darou Pakhsh Pharmaceutical Mfg.co, Iran) were dissolved or suspended in normal saline and were administered with the injection volume of 10 mL/kg while diazepam (Sigma-Aldrich, St. Louis, MO, USA) and flumazenil (Sigma-Aldrich, St. Louis, MO, USA) were dissolved in DMSO 50%. Since the DMSO has some effects on the central nervous system by itself, the injection volume for diazepam and flumazenil was 5 mL/kg. All the drugs used in the present study were injected intraperitoneally (i.p.).

*Animals*

Male NMRI mice (weighed 18 to 25 g) were used in all ‎experiments. Animals from the Animal House of Shahid Beheshti University ‎of Medical Sciences were caged in groups of ten at a controlled temperature of 22 ± 2 ºC, with free access to water and food. Animal handling in order to adaptation to the laboratory environment was done for three consecutive days before each experiment. All the procedures were conducted according to the National Institutes of Health (NIH) guidelines for the Care and Use of Laboratory Animals.

*Total phenolic content*

Total phenols were determined by colorimetric method using the Folin-Ciocalteu reagent (14, 15). To prepare a linear calibration curve, 1 mL of gallic acid standards at different concentrations (25, 50, 75, 100, 150, and 200 µg/mL; in methanol) were mixed with 5 ml of Folin–Ciocalteu reagent (diluted 1/10 with water). Five minutes later, 4 mL of aqueous Na_2_CO_3_ (75 mg/mL) was added, and the mixture was incubated at room temperature for 30 min. The same procedure was carried out on *Thymus kotschyanus* ‎extract at the concentration of 400µg/mL, and the absorbance was measured at 765 nm using a Shimadzu UV-1601PC UV-Visible spectrophotometer. This procedure was repeated in triplicate. 

**Table 1:** Total phenolic and flavonoid contents of *Thymus kotschyanus*

	**calibration curves**	**r** **2**	**Contents (µg/mg) ** **1**
Phenolic content determination	y = 0.0028x + 0.0512	0.996	225.72 ± 12.03
Flavonoid content determination	y = 0.0017x + 0.0006	0. 994	77.06 ± 4.78

1 Presented as µg of gallic acid and rutin equivalents in mg of the dry matter of the extract for total phenolic and flavonoid contents,

respectively. Values are expressed as mean ± S.E.M (n = 3).

**Table 2:** The ED50 of diazepam and the extract of *T. kotschyanus *in PTZ and MES models

**ED** **50 ** **(mg/kg) with (95% confidence interval)**		
	**PTZ**	**MES**
Diazepam	0.95 (0.64-1.31)	1.45 (0.79 - 1.76)
*Thymus kotschyanus*	424.795 (322.854-543.526)	345.395 (259.307-436.146)

**Figure 1:**
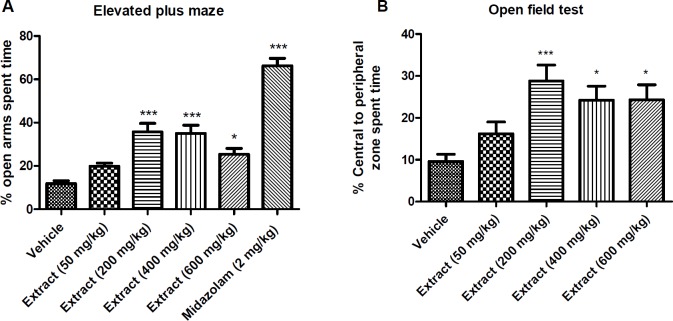
Effect of *T. kotschyanus* on the percentage of open arms spent time in EPM test (A). Effect of *T. kotschyanus* on the percentage of central to peripheral zone spent time in OFT (B). Data are presented as mean ± SEM. * indicates *p* < 0.05 and *** indicates *p* < 0.001 compared to the control group; (n = 10) in all groups

**Figure 2:**
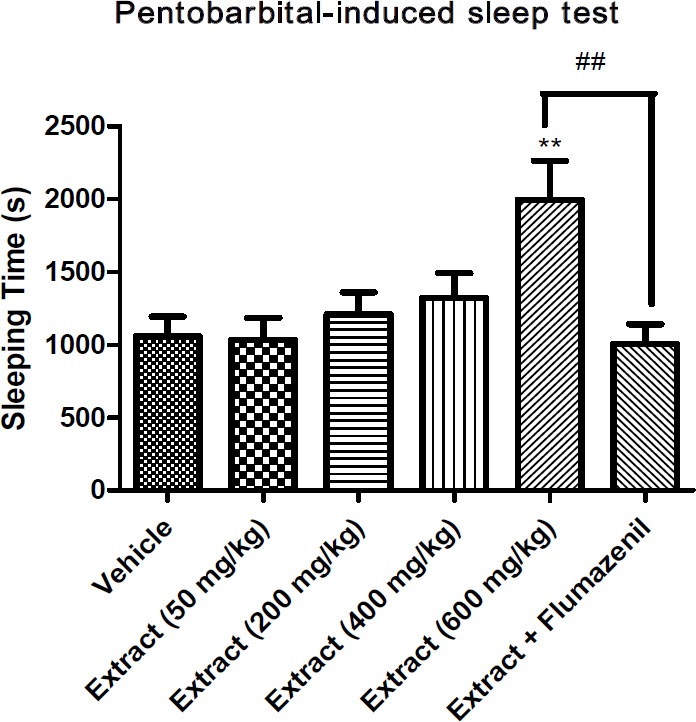
Effect of *T. kotschyanus* on potentiation of pentobarbital sleeping time. Data are presented as mean ± SEM. ** indicates *p* < 0.01 compared to the control group; ## indicates *p* < 0.01 between two compared groups; (n = 10) in all groups

**Figure 3:**
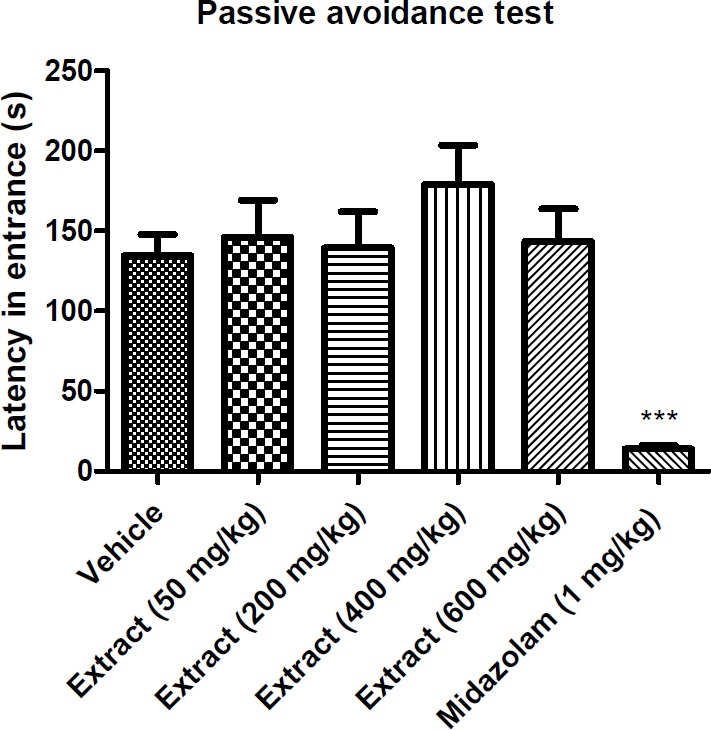
Effect of *T. kotschyanus* on avoidance latency in passive avoidance test. Data are presented as mean ± SEM. ‎*** indicates *p* < 0.001 ‎compared to the control group; (n = 10) in all groups

**Figure 4:**
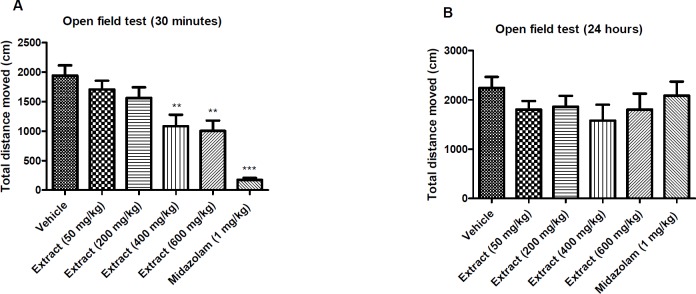
Effect of *T. kotschyanus* on the total distance movement of mice in the open field test after 30 min (A) and 24 h (B) of treatments. Data are presented as mean ± SEM. ** indicates *p* < 0.01 and ‎*** indicates *p* < 0.001 ‎compared to the control group; (n = 10) in all groups

*Total flavonoid content*

Flavonoid content was determined using Aluminum chloride reagent (14, 15). To prepare the rutin calibration curve, 2.5 mL of rutin solution at different concentrations (25, 50, 75, 100, and 150 µg/mL; in methanol) was thoroughly mixed with AlCl_3_ reagent (20 mg/mL). The same procedure was carried out on 400 µg/mL of *Thymus kotschyanus* extract. Following 40 min incubation at room temperature, the absorbance was measured at 415 nm. This procedure was repeated in triplicate. 

*Acute toxicity study*

Mice in 4 groups were treated with different doses of the extract and were observed within 72 h after each treatment. The maximum non-fatal dose and median lethal dose (LD_50_) were assumed as the dose that had not induced any mortality and the dose that caused 50% of death, respectively.

*Pentylenetetrazole and Maximal electroshock tests*

Pentylenetetrazole (PTZ) and Maximal electroshock (MES) models were used to test the anticonvulsant activity of the extract (16, 17). Mice were treated with different doses of the extract (50, 200, 400 and 600 mg/kg body weight) 30 min before induction of seizure in PTZ and MES tests. The ability of the extract to protect mice against the lethal dose of PTZ (100 mg/kg body weight) within 30 min in PTZ test and to reduce the number of hind limb tonic extension (HLTE) in mice following application of electroshock (10 Hz, 37.2 mA and 0.3 s) through the ear electrodes in MES test were assumed as the anticonvulsant activity of the extract. Diazepam (0.25, 0.5, 1 and 2 mg/kg body weight) and normal saline were used as the positive control and vehicle, respectively.

*Elevated plus maze test* *‎*

The elevated plus maze ‎(EPM) apparatus is made out of the black plexiglass and consist of two open arms (length 50 cm, width 10 cm) and two closed arms (length 50 cm, width 10 cm, and height 10 cm) extending from a central platform (10 cm × 10 cm). The plus-maze was located at a height of 50 cm above the floor level. The mice were given different doses of the extract (50, 200, 400 and 600 mg/kg body weight), vehicle and midazolam (2 mg/kg body weight) intraperitoneally and 30 min before their placement on the central platform of the EPM apparatus, facing toward one of the closed arms. Animal’s movement was recorded within 10 minutes, using a digital camera placed above the EPM apparatus. All recorded videos were analyzed by Ethovision XT (Noldus, The Netherlands) software, and the percentage of the spent time in the open arms was measured. Between each trial, the maze environment was completely cleaned by 70 % ethanol (18).

‎

*Pentobarbital-induced sleep test* *‎* ‎ 

This experiment was performed in 7 groups. Four groups received the *T. kotschyanus* extract at the doses of 50, 200, 400, and 600 mg/kg body weight, while the other groups received normal saline, diazepam (1 mg/kg body weight) and 600 mg/kg of the extract in combination with flumazenil (10 mg/kg). Thirty minutes afterward, pentobarbital sodium (40 mg/kg, body weight) was administered to each mouse for sleep induction, and the duration between loss and recovery of righting reflex (duration of sleeping) was recorded. All the experiments were performed at the same time every day to avoid the effect of circadian rhythm on the behavior of animals (19).

*Step-through passive avoidance test* ‎ ‎

In this test, the animals learned to avoid an aversive stimulus, represented by a mild foot shock. Step-through passive avoidance test was conducted in a two-compartment (20 × 20 × 20 cm each) apparatus, where one was a dark compartment and preferable to mice, and the other one was a bright compartment. The compartments were divided by a partition which had a sliding door at floor level. 

This test was conducted in two consecutive days. On the first day, the mice were treated with the extract at different doses (50, 200, 400 and 600 mg/kg body weight), vehicle and midazolam (1 mg/kg body weight). Thirty minutes later, each mouse was located in the bright compartment, facing away from the sliding door, and was allowed to explore. After 30 seconds, the sliding door was removed and when the mouse entered the dark compartment a foot shock (25 V, 0.5 mA, 2 s) was immediately delivered to the grid floor of the dark room. On the second day, this experiment was repeated while the sliding door was open and the latency to enter the dark compartment was recorded (16, 20).

*Open field test*

The open field apparatus is a cubic chamber made out of transparent Plexiglas walls (40 cm × 40 cm × 40 cm) with a digital camera placed above of it which can record the locomotion of mice. Thirty minutes and 24 h after administration of the extract, vehicle, and diazepam (2 mg/kg, body weight) each mouse was placed in the open field area for ten minutes. All recorded videos were analysed by an automated tracking system (Ethovision XT software, Noldus, The Netherlands). Ethanol (70%) and water were used to clean the open field area after every test (19).

*Statistical analysis*

Linear regression method was used for the creation of the calibration curves.‎ Probit-regression method (SPSS software, Chicago, IL; version 17.0) was used to calculate ED_50_ of the extract. The ED_50 _value is presented as mean with 95% confidence interval. One way analysis of variance and the Tukey post-test (Graph Pad Prism software, San Diego, CA; version 5.0) were used to compare differences between different groups. In all tests, *p *< 0.05 ‎was considered as the statistically significant difference.

## Results

*Acute toxicity study*

Following administration of the extract, the maximum non-fatal dose and median lethal dose (LD_50_) were 1 g/kg and 5683.8 (4763.1- 7243.9) mg/kg, respectively.

*Total phenolic and flavonoid contents*

As shown in Table 1, total phenolic and flavonoid ‎contents were calculated by using the obtained calibration curves. Results are presented as µg of gallic acid and rutin equivalents in mg of the dry matter of the extract for total phenolic and flavonoid contents, respectively.

*Anticonvulsant activity of the extract*

Pentylenetetrazole-induced seizures and maximal electroshock tests were conducted to determine the anticonvulsant activity of the extract. 

The extract of *T. kotschyanus* showed anticonvulsant activity by protection against PTZ and reduction of HLTE numbers in PTZ and MES tests. The ED_50_ values of the extract and diazepam are presented in Table 2.

*Anxiolytic activity of the extract*

The anxiolytic activity of the extract was evaluated in the elevated plus maze and open field tests (OFT). In the EPM test, the extract of *T. kotschyanus *at doses more than 50 mg/kg and midazolam at the dose of 2 mg/kg significantly increased the percentage of open arms spent time comparing to the vehicle group as shown in Figure 1-A. In the OFT, the extract of *T. kotschyanus *at doses of 200, 400 and 600 mg/kg significantly increased the percentage of central to peripheral zone spent time in the open field area and showed anxiolytic activity comparing to the vehicle group as shown in Figure 1-B.

*Hypnotic effect of the extract*

To evaluate the hypnotic effect of the extract, pentobarbital-induced sleep test was conducted. The extract at the dose of 600 mg/kg increased the sleeping time compared to the vehicle group. Flumazenil as the benzodiazepine receptor antagonist was able to significantly reduce the effect of the extract with hypnotic activity (Figure 2).

*Effect of the extract on the long-term memory of mice*

Passive avoidance test is one of the main tests used to evaluate the effect of different agents on the learning and long-term memory processes. Figure 3 shows the latency in the entrance to the dark compartment of the apparatus. The avoidance latency did not change in any group compared to the vehicle. The midazolam-treated group showed a significant reduction in latency time.

*Locomotor activity effect of the extract*

The locomotor activity of mice was analyzed in the open field test and presented as total distance movement following administration of the extract at different doses and midazolam. In the open field test, as shown in Figure 4, *T. kotschyanus* extract and midazolam apparently reduced total distance movement after 30 min (Figure 4-A), but they had no significant effect on the locomotor activity of mice after 24 h (Figure 4-B).

## Discussion

Herbal medicines are going to be more important in the treatment of patients as alternative medicines. This is because of increasing resistance to chemical drugs, toxicity, undesirable side effects and the high cost of synthetic medicines (21). *Thyme* family is well-known as one of the most important species because of its antioxidant, anti-inflammatory (22), anti-bacterial (23), antidepressant (13), anxiolytic (12), properties, and high amount of phenolic compounds (14). Although some useful properties of *T. kotschyanus* have been reported by the scientists, there is no comprehensive study on the pharmacological effects of *T.* *kotschyanus* extract on the central ‎nervous system and its toxicity profile in experimental models.

The total phenolic and flavonoid contents of *T. kotschyanus *are presented in Table 1. *T. kotschyanus *was found to have lower phenolic content and higher flavonoid content in comparison to what has been reported by Nickavar *et al*. (14). It is notable that genetic and environmental factors such as temperature, water status, light condition, nitrogen content, environmental stress, and extraction solvent can affect the total content of phenolic compounds (24). 

In order to evaluate the acute toxicity of the extract two factors including maximum non-fatal dose and median lethal dose were estimated. Since some researches (25, 26) indicate that mathematical models such as artificial neural network could be successful in ecological and biological studies, probably more information in the prediction of toxicity of the extract can be obtained from computational toxicology and mathematical modeling (27).

According to the PTZ and MES results, administration of *T. kotschyanus *showed anticonvulsant activities in both animal models used in the screening of antiepileptic agents. Interestingly, the estimated ED_50_‎_s_ are below the maximum non-fatal dose. The results of this investigation support the findings, suggesting anticonvulsant properties of *Thyme* family (28). Thymol as one of the main component of* T. kotschyanus* is associated with cessation of convulsion. Thymol is reported as a positive allosteric modulator of the GABA_A_ receptors (29, 30). It has the ability of activation of GABA_A_ receptors even in the absence of the natural agonists (31).

The results of the EPM and the OFT evoked anxiolytic activity of the extract as the mice treated with *T. kotschyanus* preferred to spend more time in open arms of EPM apparatus and showed a higher tendency to stay in the central zone of open field box. Increased anxiety has been correlated obviously with the increased reactive oxygen species (ROS) (32). Several studies have reported that anxiety-like behavior could be reversed by antioxidants (12, 33, 34). The observed anxiolytic effects could be due to phenolic monoterpenes of *T. kotschyanus* such as thymol, and carvacrol as they have been reported to possess a high antioxidant activity (35, 36). Moreover, carvacrol shows the anxiolytic effect by interaction with the dopaminergic system (37, 38).

Regarding the result of pentobarbital-induced sleep test, *T. kotschyanus *extract at the dose of 600 mg/kg showed a significant hypnotic effect compared to the control group which is probably because of interaction between phenolic compounds and α_1_-containing GABA_A_ receptors. Reversal of the hypnotic effect of the extract by flumazenil, as the GABA_A_ receptor antagonist, is the main evidence of this claim (39). This finding is in agreement with the results of previous studies that have shown the ability of thymol and some other phenolic compounds such as flavonoids to activate GABA_A_ receptors (29-31). 

Benzodiazepines are the main class of psychoactive drugs with sedative, hypnotic, anxiolytic, anticonvulsant, and muscle ‎relaxant properties which are commonly used in patients. Since benzodiazepines are indirect GABA modulators, they can enhance the effect of GABA at the GABA_A_ receptor (40). GABA_A_ ‎receptors are found to be pentameric transmembrane receptors which consist of ‎19 subunits (α_1–6_, β_1–3_, γ_1–3_, δ, ϵ, θ, π, and ρ_1–3_) (41). Recent studies on memory performance effects of benzodiazepines have revealed that interaction of these compounds with α5-containing GABA_A_ receptors can lead to altered learning performance (42). Considering the avoidance latency results in the passive avoidance test, *T. kotschyanus *treatment obviously did not impair memory function compared to the control mice. The result of open field test indicates that *T. kotschyanus* extract has a significant sedative effect by reduction of total distance movement following 30 min of exposure. Since the extract has no significant effect on the locomotor activity of mice following 24 h of exposure, it could be concluded that the results of passive avoidance test are not affected by the sedative activity of the extract. This finding reveals that the active compounds of *Thymus kotschyanus* extract more likely interact with α_1_-containing GABA_A_ receptors which are responsible for sedative-hypnotic effects of benzodiazepines, and do not bind to α_5_-containing GABA_A_ receptors which play a key role in cognitive processes.

## Conclusion

The current study showed anticonvulsant, anxiolytic, and sedative-hypnotic effects of *Thymus kotschyanus* extract in animal models. Since some phenolic compounds and especially flavonoids have been reported as herbal ligands for the benzodiazepine binding site of GABA_A_ receptors (43) and the ability of flumazenil to antagonize the observed effects, involvement of α_1_-containing GABA_A_ receptors could be reported as the possible mechanism of action, but more experiments should be carried out to find out the exact molecular mechanisms related to observed effects and the main active compound (s) which are responsible for the mentioned activities.
